# Estimates of Incidence and Predictors of Fatiguing Illness after SARS-CoV-2 Infection

**DOI:** 10.3201/eid3003.231194

**Published:** 2024-03

**Authors:** Quan M. Vu, Annette L. Fitzpatrick, Jennifer R. Cope, Jeanne Bertolli, Nona Sotoodehnia, T. Eoin West, Nikki Gentile, Elizabeth R. Unger

**Affiliations:** Centers for Disease Control and Prevention, Atlanta, Georgia, USA (Q.M. Vu, J.R. Cope, J. Bertolli, E.R. Unger);; University of Washington, Seattle, Washington, USA (A.L. Fitzpatrick, N. Sotoodehnia, T.E. West, N. Gentile)

**Keywords:** COVID-19, coronavirus disease 2019, SARS-CoV-2, severe acute respiratory syndrome coronavirus 2, viruses, respiratory infections, zoonoses, post COVID-19 conditions, PCC, post-acute sequelae of SARS-CoV-2 infection, PASC, fatigue, myalgic encephalomyelitis/chronic fatigue syndrome, ME/CFS, incidence

## Abstract

This study aimed to estimate the incidence rates of post–COVID-19 fatigue and chronic fatigue and to quantify the additional incident fatigue caused by COVID-19. We analyzed electronic health records data of 4,589 patients with confirmed COVID-19 during February 2020–February 2021 who were followed for a median of 11.4 (interquartile range 7.8–15.5) months and compared them to data from 9,022 propensity score–matched non–COVID-19 controls. Among COVID-19 patients (15% hospitalized for acute COVID-19), the incidence rate of fatigue was 10.2/100 person-years and the rate of chronic fatigue was 1.8/100 person-years. Compared with non–COVID-19 controls, the hazard ratios were 1.68 (95% CI 1.48–1.92) for fatigue and 4.32 (95% CI 2.90–6.43) for chronic fatigue. The observed association between COVID-19 and the significant increase in the incidence of fatigue and chronic fatigue reinforces the need for public health actions to prevent SARS-CoV-2 infections.

According to the Household Pulse Survey conducted by the US Centers for Disease Control and Prevention in January 2023, up to 15% of all US adults had experienced >1 symptoms of post–COVID-19 conditions (PCC), also known as long COVID or postacute sequelae of SARS-CoV-2 infection (PASC) (*1*). Among persons with PCC, fatigue is frequently reported in both hospitalized and nonhospitalized patients (*2*,*3*). A recent prospective cohort study reported 85% of patients who met its PASC definition had fatigue (*4*). A substantial percentage of patients with fatigue remain ill for many months with an illness similar to myalgic encephalomyelitis/chronic fatigue syndrome (ME/CFS) (*5*), an unexplained syndrome sometimes seen after infections that is characterized by functional limitations that impair patients’ ability to maintain daily activities and is associated with profound fatigue (*6*).

The burden, distribution, and trend of PCC can theoretically be measured by using prevalence and incidence. The prevalence of PCC is a useful measure of overall disease burden at a specific time but is dependent on recovery, deaths, and incidence. The incidence of PCC measures the rate of new cases over a certain period and can be valuable for informing public health actions to reduce new illnesses. Numerous studies have estimated PCC prevalence, but very few have attempted to estimate PCC incidence because the incidence estimate requires information on timing of incident event and a well-defined population at risk that does not include prevalent cases (*7*). Both requirements are challenging in the context of PCC because they consist of a range of conditions and symptoms, most of which are not specific to PCC. To date, no diagnostic biomarkers are available, and recognition of PCC requires integrating medical history and clinical findings. Recent studies also emphasize the importance of an equivalent, concurrent, non–COVID-19 comparison group so that the effects of COVID-19 will not be overestimated (*8*). Given the central role of fatigue in PCC and the lack of data on incidence of fatigue among patients who have had COVID-19, we conducted a study of incident fatigue diagnoses among patients with and without COVID-19. Our objectives were to estimate the incidence rates of fatigue and chronic fatigue; quantify the additional incident fatigue caused by COVID-19; assess factors associated with incident fatigue; and describe deaths and hospitalizations among patients with incident fatigue after SARS-CoV-2 infection.

## Methods

This study was designed as a retrospective cohort analysis. We analyzed electronic health records (EHR) data collected from the University of Washington (UW) that included 3 hospitals (Harborview Medical Center, UW Medical Center Northwest, and UW Medical Center Montlake) and >300 primary care and specialty clinics providing healthcare services across the state of Washington, USA.

### Case and Control Classification

COVID-19 patients consisted of adults (>18 years of age) having either a positive PCR test result for SARS-CoV-2 or a clinical diagnosis of COVID-19 during February 2020–February 2021 (*9*). A clinical diagnosis of COVID-19 was defined by an International Classification of Diseases, 10th Revision, Clinical Modification (ICD-10-CM), diagnostic code of B97.29, other coronavirus as the cause of diseases classified elsewhere; or U07.1, COVID-19, recorded in the EHR during February 2020–February 2021 (*10*). The index date was defined as the date of the first positive PCR result or the first clinical diagnosis, whichever was earlier.

Non–COVID-19 control patients were defined as adults who did not belong to the COVID-19 group and had >1 negative PCR for SARS-CoV-2 during February 2020–February 2021. The first negative test date is referred to as the index date. We excluded from this group persons with suspected COVID-19 or evidence of past COVID-19, including persons with any of the following ICD-10-CM codes: B34.2, coronavirus infection, unspecified; J12.82, pneumonia due to COVID-19; Z86.16, personal history of COVID-19; U09.9, post COVID-19 condition. We also excluded persons with a positive result on SARS-CoV-2 IgG.

### Inclusion and Exclusion Criteria

**Figure 1:**
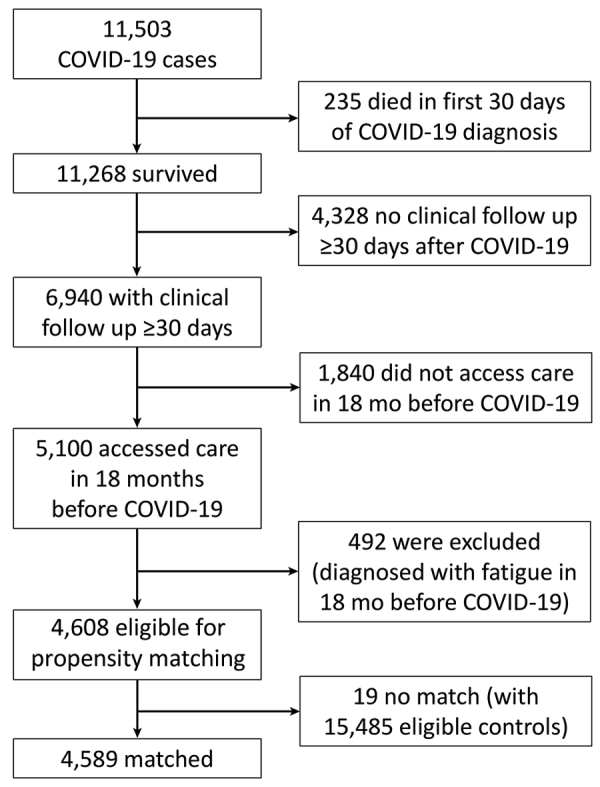
Data flow for COVID-19 cases in study of incidence and predictors of fatiguing illness after SARS-CoV-2 infection, Washington, USA, February 2020–February 2021.

Patients in both COVID-19 case and non–COVID-19 control groups were required to survive the first 30 days from index date; access care >1 time on or after the day 30 from the index date, defined by having a diagnosis code or a laboratory test; access care >1 time during the 18 months before the index date for evaluation of preexisting fatigue diagnoses; and not be diagnosed with any codes used to define fatigue during the 18 months before the index date. During February 2020–February 2021, a total of 11,503 unique patients received a COVID-19 diagnosis. A total of 4,608 COVID-19 patients were eligible for matching (Figure 1).

We extracted data from 15,834 non–COVID-19 patients by querying the study database using the previously described inclusion and exclusion criteria, as well as the same requirements for accessing care. After data cleaning, 15,485 non–COVID-19 patients were determined to be eligible for matching.

### Propensity Score Matching

We used propensity score matching to achieve balance in selected characteristics for COVID-19 and non–COVID-19 groups (*11*). We estimated propensity score using logistic regression with 22 input variables of age, sex, race, ethnicity, and whether the person had comorbidities derived from the Charlson Comorbidity Index (CCI) during the 18-month period before the index date (Table 1) (*12*). We then matched patients on the logit of propensity score using the greedy method with a caliper of 0.2 SD of the logit of the score.

**Table 1:** Characteristics of patients with COVID-19 and matched controls in study of incidence and predictors of fatiguing illness after SARS-CoV-2 infection, Washington, USA, February 2020–February 2021*

Description	Patients, n = 4,589	Controls, n = 9,022
Age, y, mean (SD)	49.5 (17.8)	49.0 (18.0)
Sex		
F	2,248 (49.0)	4,447 (49.3)
M	2,341 (51.0)	4,575 (50.7)
Race		
Asian	418 (9.1)	807 (8.9)
Black	704 (15.3)	1136 (12.6)
Indian/Alaska native	97 (2.1)	153 (1.7)
Native Hawaiian Pacific	82 (1.8)	119 (1.3)
White	2,942 (64.1)	5,825 (64.6)
Missing	346 (7.5)	982 (10.9)
Ethnicity		
Hispanic/Latino	613 (13.4)	1,166 (12.9)
Not Hispanic/Latino	3,709 (80.8)	7,033 (78.0)
Missing	267 (5.8)	823 (9.1)
Underlying conditions†		
Acute myocardial infarction	90 (2.0)	147 (1.6)
History of myocardial infarction	97 (2.1)	149 (1.7)
Congestive heart failure	289 (6.3)	490 (5.4)
Peripheral vascular disease	257 (5.6)	451 (5.0)
Cerebrovascular disease	231 (5.0)	410 (4.5)
COPD	667 (14.5)	1,259 (14.0)
Dementia	73 (1.6)	122 (1.4)
Hemiplegia or paraplegia	99 (2.2)	178 (2.0)
Diabetes	678 (14.8)	1,262 (14.0)
Diabetes with complications	354 (7.7)	635 (7.0)
Moderate–severe renal disease	400 (8.7)	715 (7.9)
Mild liver disease	318 (6.9)	559 (6.2)
Moderate–severe liver disease	46 (1.0)	81 (0.9)
Peptic ulcer disease	43 (0.9)	71 (0.8)
Rheumatologic disease	81 (1.8)	128 (1.4)
HIV/AIDS	129 (2.8)	218 (2.4)
Any malignancy, except skin	382 (8.3)	677 (7.5)
Metastatic solid tumor	111 (2.4)	193 (2.1)

*Values are no. (%) except as indicated. Race includes no information on Hispanic ethnicity. COPD, chronic obstructive pulmonary disease. †Diagnosed in the 18 mo before date of COVID-19 confirmation or date of negative test.

Among 4,608 patients with COVID-19 who were eligible for matching, 19 (0.4%) had no matched controls. Among 4,589 patients with COVID-19 who had >1 match with 9,022 non–COVID-19 controls, 4,433 patients (96.6%) had 2 matched controls and 156 (3.4%) had 1. After matching, the standardized differences for 22 input variables used for estimating propensity score and for the index date were all <0.1, indicating between-group balances in these variables (*13*).

### Outcome Measures

Outcome events of interest were patients with >1 diagnostic codes for fatigue or chronic fatigue recorded in the EHR during the postacute period. The postacute period was defined as the time between the 30th day since the index date and the last follow-up date up to January 2022.

Fatigue was defined by any of the following ICD-10-CM or International Classification of Diseases, 9th Revision, Clinical Modification (ICD-9-CM), diagnostic codes recorded in EHR during the postacute period: G93.3, postviral fatigue syndrome; R53.82, chronic fatigue, unspecified; R53.83, other fatigue; 780.71, chronic fatigue syndrome/postviral fatigue syndrome; or 780.79, malaise and fatigue. We defined incident fatigue as a patient who had >1 diagnostic code for fatigue during the postacute period.

In this study, chronic fatigue is a subset of fatigue, defined as having any of the following 3 ICD-10-CM or ICD-9-CM codes recorded in the EHR during the postacute period: G93.3, postviral fatigue syndrome; R53.82, chronic fatigue, unspecified; and 780.71, chronic fatigue syndrome/postviral fatigue syndrome. We defined incident chronic fatigue as a patient who had >1 diagnostic code for chronic fatigue during the postacute period.

### Follow-Up Time and Censoring

The last follow-up date was defined as the death date or the last date of having a clinical diagnosis or laboratory test up to January 2022. The follow-up time was calculated as time from the index date to the date of the first incident event for patients with an event, or as time from the index date to the last follow-up date for those without an event (right censoring).

### Statistical Methods

We estimated incidence rates of fatigue and chronic fatigue for COVID-19 case and non–COVID-19 control groups using frequencies of events during the follow-up time, assuming a Poisson distribution of events. To quantify the attribution of COVID-19 to fatigue and chronic fatigue diagnoses, we used proportional hazards models that employed robust variance estimators to adjust for dependences associated with matching (*14*).

To examine potential predictors of incident fatigue among 4,589 patients with COVID-19, we used the Clinical Classifications Software Refined to aggregate diseases and conditions diagnosed within 18 months before COVID-19 into clinically meaningful categories (*15*). We analyzed data for categories with prevalence >1% and used the log-rank test to compare survival functions for each of the categories. We used multivariable proportional hazards models to identify factors associated with incident fatigue, adjusting for age, sex, and total number of comorbidities derived from the CCI (*12*,*16*). To assess the assumption of proportional hazards, we generated time-dependent covariates as a function of the predictors and follow-up time then evaluated the covariates in the model. We used proportions and crude relative risk (RR) to compare proportions of deaths and hospitalizations among patients with COVID-19 with fatigue versus those without fatigue. We performed all analyses using SAS 9.4 (SAS Institute, Inc., https://www.sas.com).

### Human Subjects Considerations

This analysis is part of Project RELIEF (Research on COVID-19 Long-Term Effects). This activity was reviewed by the Centers for Disease Control and Prevention and was conducted consistent with applicable federal law and center policy. All protocols, procedures, and consent processes used in Project RELIEF were reviewed and approved by the University of Washington Institutional Review Board Committee A (STUDY00014595).

## Results

### Patients

The study population had a mean age of 49.5 years for cases and 49.0 years for controls (Table 1). Approximately half of the patients were women. The most common comorbidities were diabetes and chronic obstructive pulmonary disease, each with 14% prevalence. Approximately 55% of the population had no comorbidities, and 6% had 4–10 comorbidities derived from the CCI.

### Fatigue

During the total of 4,241.9 person-years of follow-up of 4,589 COVID-19 cases (median 11.4 months, range 1–21.4 months), 434 (9.5%) incident fatigue cases were identified, resulting in an incidence rate of 10.2/100 person-years. Of the 434 case-patients, 241 (55.5%) were women, the mean age was 52.6 (SD 17.3) years, and 165 (38.0%) patients did not have comorbidities.

The incidence rate of fatigue diagnosis was higher among women than among men and increased with advancing age (Table 2). We noted no strong evidence of a racial or ethnic difference in incidence of fatigue, except a slightly lower incidence among Black patients. Persons with more comorbidities experienced higher incidence rates than did persons without comorbidities. However, even among younger persons (18–29 years of age), those without comorbidities, and those who were not hospitalized for acute COVID-19, the incidence of fatigue was only slightly reduced (7.3/100 person-years for younger persons, 7.4/100 person-years for persons without comorbidities, and 9.9/100 person-years for persons who were not hospitalized).

**Table 2:** Incidence rate of fatigue among patients with COVID-19 in study of incidence and predictors of fatiguing illness after SARS-CoV-2 infection, by selected characteristics, Washington, USA, February 2020–February 2021*

Description	No. (%) patients	Incidence rate/100 person-years			Proportional hazards model	
		Estimate (95% CI)	p value		HR (95% CI)	aHR (95% CI)
All patients	4,589 (100.0)	10.2 (9.3–11.2)				
Sex						
F	2,248 (49.0)	11.6 (10.2–13.1)	<0.01		1.29 (1.07–1.56)	1.39 (1.15–1.69)
M	2,341 (51.0)	9.0 (7.8–10.3)	Referent		Referent	Referent
Age group, years						
18–29	771 (16.8)	7.3 (5.5–9.7)	Referent		Referent	Referent
30–59	2,344 (51.1)	10.3 (9–11.7)	0.03		1.39 (1.02–1.90)	1.23 (0.90–1.69)
>60	1,474 (32.1)	11.6 (9.9–13.5)	<0.01		1.56 (1.13–2.14)	1.21 (0.86–1.69)
Race						
Asian	418 (9.1)	11.1 (8.2–15)	0.93		1.02 (0.74–1.41)	
Black	704 (15.3)	7.8 (5.9–10.2)	0.03		0.71 (0.53–0.96)	
American Indian/Alaska Native	97 (2.1)	15.4 (9.1–25.9)	0.21		1.41 (0.83–2.41)	
Native Hawaiian/Pacific Islander	82 (1.8)	6.3 (2.6–15)	0.22		0.56 (0.23–1.37)	
White	2,942 (64.1)	10.9 (9.8–12.2)	Referent		Referent	
Missing	346 (7.5)	7.5 (4.9–11.4)	0.09		0.69 (0.45–1.06)	
Ethnicity						
Hispanic/Latino	613 (13.4)	11.2 (8.8–14.4)	0.54		1.09 (0.83–1.42)	
Not Hispanic/Latino	3,709 (80.8)	10.3 (9.3–11.5)	Referent		Referent	
Missing	267 (5.8)	6.2 (3.7–10.5)	0.06		0.61 (0.36–1.04)	
Hospitalized first 30 d						
Yes	689 (15.0)	12.1 (9.6–15.2)	0.13		1.22 (0.95–1.57)	
No	3,900 (85.0)	9.9 (9.0–11.0)	Referent		Referent	
No. underlying conditions†						
0	2,511 (54.7)	7.4 (6.3–8.6)	Referent		Referent	Referent
1–3	1,780 (38.8)	12.9 (11.3–14.7)	<0.01		1.73 (1.42–2.12)	1.73 (1.40–2.13)
4–10	298 (6.5)	16.4 (12.3–21.9)	<0.01		2.21 (1.59–3.06)	2.30 (1.63–3.24)

*Blank cells in aHR column indicate variables not included in multivariable model. aHR, adjusted HR, obtained from multivariable proportional hazards model; HR, hazard ratio, obtained from simple proportional hazards model. †Any of the following conditions diagnosed within 18 mo before COVID-19: acute myocardial infarction, history of myocardial infarction, congestive heart failure, peripheral vascular disease, cerebrovascular disease, chronic obstructive pulmonary disease, dementia, hemiplegia or paraplegia, diabetes, diabetes with complications, moderate–severe renal disease, mild liver disease, moderate–severe liver disease, peptic ulcer disease, rheumatologic disease, HIV/AIDS, any malignancy except skin, metastatic solid tumor.

**Figure 2:**
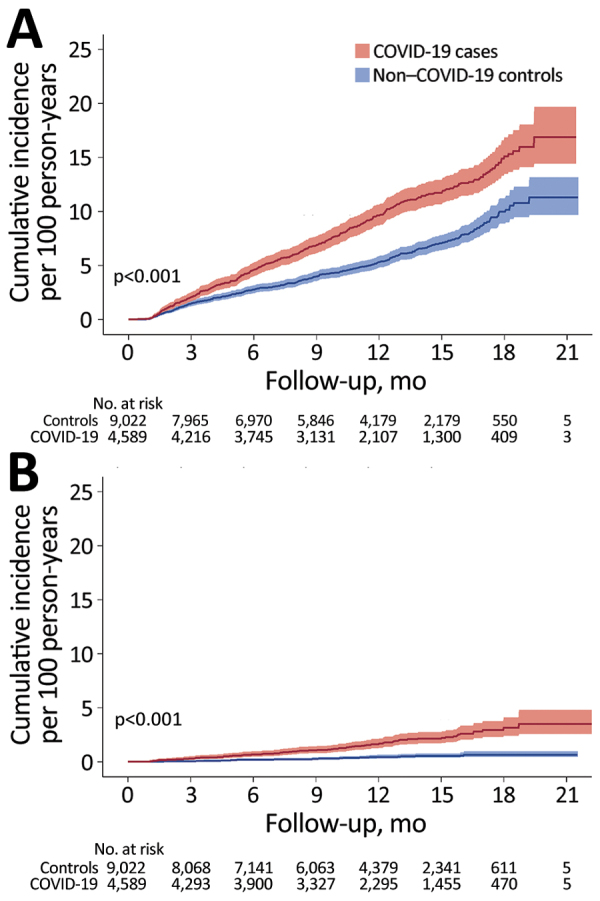
Cumulative incidence of fatigue (A) and chronic fatigue (B) among 4,589 COVID-19 cases and 9,022 non–COVID-19 controls in study of fatiguing illness after SARS-CoV-2 infection, Washington, USA, February 2020–February 2021. Shading around data lines indicates 95% CIs.

During the total of 7,939.1 person-years of follow-up of 9,022 non–COVID-19 controls (median 11.5 months, range 1–21.5 months), we identified 477 incident fatigue cases, resulting in an incidence rate of 6.0/100 person-years. The risk of incident fatigue was 68% higher among COVID-19 cases than among non–COVID-19 controls (hazard ratio 1.68, 95% CI 1.48–1.92; p<0.001) (Figure 2, panel A).

### Chronic Fatigue

We next examined the incidence of chronic fatigue diagnosis, a subset of fatigue. During follow-up, 81 COVID-19 patients received a diagnosis of incident chronic fatigue, resulting in an incidence rate of 1.82 (95% CI 1.47–2.27)/100 person-years. The incidence rate of chronic fatigue among non–COVID-19 controls was 0.42 (95% CI 0.29–0.58)/100 person-years. The risk of developing chronic fatigue was significantly higher for COVID-19 cases compared with non–COVID-19 controls (HR 4.32, 95% CI 2.90–6.43; p<0.001). The difference between cumulative incidence for COVID-19 patients and non–COVID-19 controls continued to increase without apparent plateau >12 months after the index date (Figure 2, panel B).

### Predictors of Incident Fatigue

Women were 39% more likely to have a fatigue diagnosis than men were after adjusting for age group and comorbidities (Table 2). Persons of advancing age groups were more likely than young adults 18–29 years of age to have a fatigue diagnosis in an unadjusted model. After adjusting for sex and comorbidities, the HRs for advancing age groups were still elevated, but the differences were no longer statistically significant. Those with comorbidities were significantly more likely to have incident fatigue compared with those with no comorbidities.

Among 36 diseases and conditions diagnosed in the 18 months before COVID-19 with a prevalence ≥1% that show difference in incident fatigue (log-rank p<0.05), 21 conditions remained associated (p<0.05) with incident fatigue when each was included in a multivariable proportional hazards model that adjusted for age, sex, and number of comorbidities. Obesity was associated with incident fatigue in the simple model, but the association became nonsignificant in the adjusted model. The risk for incident fatigue that was significantly higher for other diseases and conditions (Table 3) ranged from 27% increased risk for persons with hypertension to 93% increased risk for persons with gastritis and duodenitis.

**Table 3:** Associations between incident fatigue and diseases and conditions diagnosed in 18 months before SARS-CoV-2 infection among 4,589 patients with COVID-19 in study of incidence and predictors of fatiguing illness after SARS-CoV-2 infection, Washington, USA, February 2020–February 2021*

Description	Proportional hazards model				
	Simple			Multivariable†	
	HR (95% CI)	p value		aHR (95% CI)	p value
Circulatory system					
Essential hypertension	1.53 (1.27–1.86)	<0.001		1.27 (1.01–1.59)	0.043
Digestive system					
Biliary tract disease	2.27 (1.43–3.59)	<0.001		1.71 (1.06–2.74)	0.027
Gastroesophageal reflux disease and other esophageal disorders	1.53 (1.23–1.90)	<0.001		1.29 (1.02–1.62)	0.032
Gastritis and duodenitis	2.10 (1.38–3.20)	<0.001		1.93 (1.26–2.94)	0.002
Endocrine					
Hypothyroidism and other thyroid disorders	1.84 (1.41–2.39)	<0.001		1.44 (1.09–1.89)	0.011
Nutritional deficiency, including vitamin D, B, iron	1.86 (1.35–2.56)	<0.001		1.55 (1.12–2.15)	0.008
Obesity	1.55 (1.19–2.02)	0.001		1.22 (0.93–1.61)	0.156
Musculoskeletal system and connective tissue					
Low back pain	1.60 (1.27–2.01)	<0.001		1.42 (1.13–1.79)	0.003
Musculoskeletal pain, not low back pain	1.74 (1.44–2.10)	<0.001		1.58 (1.31–1.92)	<0.001
Osteoarthritis	1.88 (1.46–2.41)	<0.001		1.61 (1.23–2.09)	<0.001
Neoplasms					
Neoplasms of unspecified nature or uncertain behavior	2.2 (1.56–3.11)	<0.001		1.87 (1.31–2.66)	<0.001
Nervous system					
Headache, including migraine	1.82 (1.33–2.49)	<0.001		1.67 (1.22–2.29)	0.002
Nerve and nerve root disorders	1.91 (1.31–2.78)	<0.001		1.74 (1.19–2.53)	0.004
Nervous system pain and pain syndromes	1.61 (1.30–1.99)	<0.001		1.39 (1.12–1.74)	0.003
Sleep disorders	1.85 (1.49–2.28)	<0.001		1.59 (1.27–1.99)	<0.001
Psychiatry					
Anxiety and fear-related disorders	1.68 (1.35–2.10)	<0.001		1.57 (1.25–1.97)	<0.001
Depressive disorders	1.82 (1.47–2.25)	<0.001		1.62 (1.30–2.01)	<0.001
Trauma- and stressor-related disorders	1.59 (1.16–2.17)	0.004		1.46 (1.07–2.00)	0.018
Otolaryngology					
Otitis media	1.89 (1.04–3.44)	0.037		1.84 (1.01–3.34)	0.047
Respiratory system					
Acute upper respiratory infection	1.63 (1.30–2.04)	<0.001		1.62 (1.29–2.03)	<0.001
Allergic rhinitis	2.01 (1.50–2.71)	<0.001		1.80 (1.33–2.43)	<0.001
Sinusitis	1.55 (1.07–2.25)	0.021		1.56 (1.08–2.26)	0.019

*Reference for all categories is patients without the given disease/condition. Underlying conditions: acute myocardial infarction, history of myocardial infarction, congestive heart failure, peripheral vascular disease, cerebrovascular disease, chronic obstructive pulmonary disease, dementia, hemiplegia or paraplegia, diabetes, diabetes with complications, moderate-severe renal disease, mild liver disease, moderate-severe liver disease, peptic ulcer disease, rheumatologic disease, HIV/AIDS, any malignancy except skin, metastatic solid tumor. aHR, adjusted HR; HR, hazard ratio. †Multivariable proportional hazards regression models adjusting for age, sex, and number of underlying conditions, unless otherwise noted.

### Deaths and Hospitalizations

**Figure 3:**
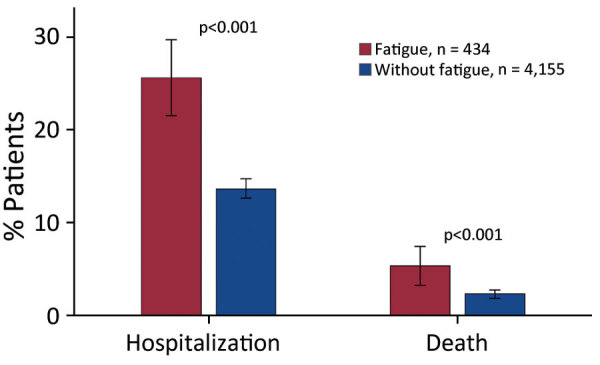
Clinical outcomes among COVID-19 patients with and without incident fatigue after SARS-CoV-2 infection in study of fatiguing illness after SARS-CoV-2 infection, Washington, USA, February 2020–February 2021.

Patients with COVID-19 in whom incident fatigue developed had far worse clinical outcomes, as evidenced by deaths and hospitalizations, than patients without fatigue (Figure 3). Among 434 COVID-19 patients in whom fatigue developed, 111 (25.6%) were hospitalized >1 time during the postacute period, whereas 13.6% of 4,155 patients without incident fatigue were hospitalized (RR 1.88, 95% CI 1.57–2.24; p<0.001). Moreover, COVID-19 patients with incident fatigue were at higher risk of dying (23/434, 5.3%) during the postacute period than were COVID-19 patients without incident fatigue (94/4,155 [2.3%]; RR 2.34, 95% CI 1.50–3.66; p<0.001).

## Discussion

In this community-based cohort study of >4,500 adults followed for an average of 11.4 months after COVID-19 infection, fatigue developed in 9%. Even among persons not hospitalized for acute COVID-19 or those without comorbidities, the incidence of post–COVID-19 fatigue approached 10% per year. COVID-19 patients had 1.68 times the risk for fatigue in the follow-up period compared with concurrent, matched non–COVID-19 controls. The risk for chronic fatigue was even more marked: patients with COVID-19 had 4.32 times the risk for chronic fatigue than did controls.

This study provides new estimates of the incidence rate of fatigue using person-years of follow-up of at-risk patients after COVID-19 infection. Our data can be put in the context of previous reports. A retrospective study of EHR data reported 12.8% of patients had received a diagnosis of incident fatigue within 6 months of COVID-19 infection (*17*). That report had a different follow-up time and did not describe whether preexisting fatigue cases were excluded from the incident fatigue counts, which might explain their higher proportion than our estimate of 9.5%. In another retrospective study of insurance claims where preexisting fatigue diagnoses were excluded from the incident event count, 4.6% of COVID-19 patients received a diagnosis of fatigue during the follow-up of <6 months (*18*). That proportion approaches our estimate of 5% cumulative incidence of fatigue for 6 months.

An incidence rate of 4.2/100 person-years for post–COVID-19 fatigue was reported from Germany (*19*). That study counted cases occurring from 3 months after infection, which potentially contributed to lower event counts. Of note, follow-up times for patients with an incident event were assigned on the basis of the calendar quarter of the insurance claim submission, and the follow-up times for patients without events were not described. A combination of those methodological differences might have contributed to the lower incidence estimate in that study.

The excess risk for fatigue attributable to COVID-19 estimated in our study is in range of previous estimates. Specifically, our hazard ratio for fatigue of 1.68 (95% CI 1.48–1.92) indicates that when compared with a concurrent control population without COVID-19, COVID-19 contributes to a 68% increase in the rate of incident fatigue. This finding mirrors the previous estimates in studies using EHR data (HR 1.65) or administrative claims data (HR 2.20, 95% CI 1.48–3.27) in the United States or in Germany (incidence rate ratio [IRR] 1.97, 95% CI 1.89–2.06) (*17*–*19*).

This study also provides new estimates of incidence rate of chronic fatigue, including ME/CFS after COVID-19 illness. The incidence rate of 1.8/100 person-years is notable, as is the observation that chronic fatigue diagnoses continued in the 18 months of follow-up after COVID-19 detection. The extended period of incident chronic fatigue occurrences suggests a persistent effect but could also indicate a delay in diagnosing fatigue as a separate symptom or diagnosis. The hazard ratio for chronic fatigue (4.32, 95% CI 2.90–6.43) indicates that COVID-19 illness results in 4.3 times the risk for chronic fatigue compared with non–COVID-19 group. That increase is similar to findings from a study of chronic fatigue syndrome in Germany (IRR 3.04, 95% CI 2.66–3.48) (*19*). Although chronic fatigue is not the same as chronic fatigue syndrome or ME/CFS, which requires additional symptoms for diagnosis, including activity limitation, postexertional malaise, unrefreshing sleep, and either cognitive impairment or orthostatic intolerance (*20*), the ICD-9 and ICD-10 codes used for the diagnosis of ME/CFS were included in the diagnostic codes used to define the chronic fatigue diagnosis. The recently implemented diagnostic code G93.32 for ME/CFS when used in conjunction with code U09.9, post COVID-19 condition, will be instrumental in identifying COVID-19–related ME/CFS in future research (*21*).

We found many diseases and conditions to be associated with post–COVID-19 fatigue. Those associations might provide useful prognostic information for the assessment of patients with COVID-19. Patients with mood disorders were previously reported to be at higher risk for illness and death during acute COVID-19 and increased risk of needing postacute care (*22*). Our findings indicate that patients with a history of mood disorders are also at increased risk for post–COVID-19 fatigue. The association of post–COVID-19 fatigue with pain syndromes and sleep disorders is supported by previous research in non–COVID-19 populations (*23*).

Our study has several strengths, including addressing a critical data gap in incidence measure of post–COVID-19 fatigue; robust application of cohort methodologies in incidence estimation using EHR data; that the EHR data were collected from a comprehensive, multiclinic, multihospital health system; a well-defined population at risk for identifying the incident event; and the rigorous selection of concurrent non–COVID-19 matched controls. However, several limitations deserve consideration. First, because we used EHR data for this study, our findings apply only to patients who access care. Future studies are needed to understand the incidence of post-COVID fatigue among those who do not access care, which would likely require different methods. Second, data on exact date of onset, duration, and severity of fatigue or related functional limitations are unavailable for further characterization. The date of fatigue documented in EHR does not necessarily represent the date of symptom onset. In addition, providers might continue to document fatigue or carry forward the diagnosis. Therefore, relying on coding for chronic fatigue without an exact date of symptom onset might underestimate incidence of chronic fatigue. Moreover, the sense of fatigue is subjective and can be underrecorded if it is being considered as part of a disease process. The introduction of code U09.9, post COVID-19 condition, in October 2021 would not change results because it would need to be coded in conjunction with fatigue. Third, data on COVID-19 vaccination were not recorded for most patients, precluding further analysis. Fourth, the relatively small number of patients with fatigue who experienced hospitalization or death during follow-up precluded further multivariable analyses to adjust for potential confounders. The unadjusted association between fatigue and hospitalization or death might have been the result of the greater comorbidities seen in persons with fatigue. Fifth, this article is focused on post–COVID-19 fatigue, but PCC is generally experienced with multisystem symptom clusters. This study was not designed to capture symptom clusters, such as postexertional malaise or symptoms other than fatigue that might also be associated with subsequent outcomes. Last, our data were limited to persons who were tested or received a diagnosis in the first 13 months of the pandemic in Washington, which was 3 months before the Delta variant was detected and 9 months before Omicron was detected (*24*). Early research indicates that the prevalence of post–COVID-19 fatigue was similar across pre-Delta variants, Delta variants, and Omicron variants, but the prevalence of severe fatigue after infections with pre-Delta variants was slightly higher than for other variants (*25*). Future research is needed to estimate incidence rates of fatigue after infections with Delta and Omicron variants and compare them with the findings from this study.

In our unadjusted analyses, patients with COVID-19 who had incident fatigue were at higher risk for hospitalization and death than were persons without incident fatigue. The severe outcome is likely driven, at least in part, by some of the comorbidities and predictors identified in this study. Elevated death rate was previously reported among fatigued patients without COVID-19 (HR 1.45) (*26*). Increased awareness of fatigue and other PCC is warranted to enable patients to seek early care when needed. Further research is also warranted to investigate the causes and preventive measures for the severe outcomes associated with post-COVID fatigue.

In conclusion, our data indicate that COVID-19 is associated with a significant increase in new fatigue diagnoses, and physicians should be aware that fatigue might occur or be newly recognized >1 year after acute COVID-19. Future study is needed to better understand the possible association between fatigue and clinical outcomes. The high incidence rates of fatigue reinforce the need for public health actions to prevent infections, to provide clinical care to those in need, and to find effective treatments for post–acute COVID-19 fatigue. 
